# Computational tool choice impacts CRISPR spacer-protospacer detection

**DOI:** 10.1093/bioinformatics/btag394

**Published:** 2026-06-29

**Authors:** Uri Neri, Antonio Pedro Camargo, Brian Bushnell, Rick Beeloo, Simon Roux

**Affiliations:** Department of Energy, Joint Genome Institute, Berkeley, CA 94720, United States; Department of Energy, Joint Genome Institute, Berkeley, CA 94720, United States; Department of Energy, Joint Genome Institute, Berkeley, CA 94720, United States; Department of Biology, Utrecht University, Utrecht, 3584, The Netherlands; Department of Energy, Joint Genome Institute, Berkeley, CA 94720, United States

## Abstract

**Motivation:**

CRISPR spacer-protospacer matching is widely used to infer host–virus interactions in microbial and viromics studies, but the choice of sequence search or alignment tool and its reporting behavior is often under-evaluated for this specific task.

**Results:**

Using synthetic, semi-synthetic, and real datasets, we benchmarked commonly used tools and observed substantial differences in recall, runtime, and resource usage across distance metrics and thresholds. Our analyses support practical defaults for large-scale spacer-target matching and clarify trade-offs between exhaustive and heuristic approaches.

**Availability:**

Source code and benchmark workflows are available at https://github.com/UriNeri/spacer_matching_bench. Data and run artifacts are archived on Zenodo (https://doi.org/10.5281/zenodo.15171878).

## Introduction

CRISPR (clustered regularly interspaced short palindromic repeats) systems play a vital role in prokaryotic defense against mobile genetic elements, including viruses, plasmids, and other autonomous genetic elements (Mojica *et al.* 2005, Koonin *et al.* 2022). These systems are organized as arrays in the bacteria or archaea genome, where short sequences called spacers are interspersed between repeated sequences. The spacer sequences within these arrays guide the targeting of invasive genetic elements, allowing for specific defense against these threats (Makarova *et al.* 2020). The corresponding locus on the virus genome which the spacer complements is termed “protospacer.” Beyond their natural role in prokaryotic immunity, CRISPR systems have been adapted into powerful gene-editing frameworks for biotechnology and therapeutic applications (Jiang and Doudna 2017), though the computational considerations for analyzing natural CRISPR-mediated phage–host interactions differ substantially from predicting off-target effects in gene editing contexts.

The analysis of spacer-protospacer pairs is essential in understanding the complex interactions between hosts and MGEs (Edwards *et al.* 2016). However, the identification of genuine host–MGE interactions through spacer-protospacer matching presents unique challenges due to the dynamic nature of these relationships and the complexity of sequence evolution. While matches between spacers and protospacers are often interpreted as evidence of interaction, various biological and technical factors can complicate this interpretation (Edwards *et al.* 2016, Soto-Perez *et al.* 2019).

Several key scenarios can lead to false positive assignments in spacer-protospacer matching. Low complexity sequences can create spurious matches between simple repeat regions [albeit these can be mitigated through complexity filtering such as tantan (Frith 2011) or DUST (Morgulis *et al.* 2006)]. Another type of potential false positives are highly conserved sequences shared by unrelated MGEs, potentially resulting from horizontal gene transfer between MGEs. The horizontal transfer of CRISPR arrays themselves on mobile elements further requires careful examination of array genomic context (regions outside the CRISPR loci) and phylogenetic analysis. Self-targeting events, where matches occur against the host genome rather than MGEs, necessitate comparison against host genome databases and analysis of targeting context (Stern *et al.* 2010). Finally, historical acquisition events may not reflect current interactions, requiring consideration of phylogenetic dating, evolution rates and the effects of the protospacers being under selective pressure to mutate (which may enable the MGE to escape the CRISPR system). This is further complicated by the fact that increasing the allowed distance between sequences directly increases the likelihood of identifying non-related sequences as similar (sharing high nucleic identity) to each other.

False negatives present another challenge in spacer-protospacer matching, particularly when dealing with large databases of potential targets. Many alignment and search tools default to reporting only the best (top) matches or the first matches that pass a given threshold for a given query or HSP. In the context of spacer-target matching, this may result in potentially missing additional legitimate matches. Unfortunately, different tools also handle ambiguous or secondary alignments differently: they may be reported completely, reported up to a number or based on relative alignment quality, or omitted. Similarly, cases where a query sequence has multiple equally scoring matches in different reference sequences are not handled uniformly across tools. This limitation becomes increasingly problematic as databases grow larger and more diverse, a single spacer might match (implying a targeting) multiple related MGEs.

Yet despite these variations, the choice of spacer-to-protospacer search or alignment tool is often not deeply considered. Presently, the common option for this task, popularized by Edwards *et al.* (2016) and Biswas *et al.* (2013), uses BLASTn (Altschul *et al.* 1990) with parameters adjusted for short input sequences. Biswas used BLASTn specifically with the -ungapped flag and used a scoring system to keep total mismatch number ≤3. However as for most bioinformatic tools, the exact workflow design and parameter choice can impact the outcome. The importance of proper tool usage and parameter interpretation is highlighted by historical examples in bioinformatics. A striking example is the work of Shah *et al.* (2019), in which they report how certain misunderstandings of BLAST’s -max_target_seqs parameter may lead to incorrect assumptions about result completeness, potentially impacting published analyses. This specific case was later clarified by Madden *et al.* (2019) (of the blast development team) as an unfortunate combination of a software bug (that were since patched) affecting rare cases, and misconceptions regarding the process BLAST+ uses for tie-breaking (alignments of equal plausibility), and finally a consideration regarding composition base scoring. Apart from the patched bug, the main outcome of this correspondence led to more explicit details in blast documentation (specifically the appendix “Outline of the BLAST process”). Still, this highlights that misconceptions about the expected exhaustiveness of tools’ result-reporting can also lead to incorrect assumptions about the outcome of an analysis. In practice, most bioinformatic tools use various heuristics and optimizations, typically designed with specific use cases in mind. For example, most short-read mappers assume the reference to be the output of a singular assembly—which would imply the reference does not contain redundant copies of the same nucleic regions, or a limited number of very similar sequences (e.g. strain variants, alternative splice variants), and this assumption impacts the way read mapping is computed and results are reported.

The choice of tool and its parameters can significantly impact the detection of these multiple matches, with some tools prioritizing speed over completeness by limiting the number of reported matches, or by other internal heuristics such as seed sequence selection from high occurring sequences being penalized. This trade-off between sensitivity and computational efficiency is especially important to consider as most available tools were designed for different tasks than spacer-protospacer matching (e.g. expression analysis, homology detection, and variant calling), and under different assumptions (such as reference and query sequence size and database size or the nature of the reference source: from a single isolate or metagenomic sample rather than from aggregation of sequences from different sources).

### Computational foundations of sequence similarity

From a computer science perspective, biological sequences are represented as strings of characters drawn from finite alphabets: DNA and RNA sequences use the four-letter nucleobase alphabet (A, U/T, G, C), while protein sequences use the twenty-letter amino acid alphabet. Determining sequence similarity thus becomes a string-matching problem, where the goal is to find all occurrences of a query string (or similar variants) within a reference string or database, subject to specified constraints on permitted differences. The fundamental challenge lies in defining (and efficiently computing) a meaningful notion of “similarity” between sequences that may have diverged through evolutionary processes including substitutions, insertions, deletions, and rearrangements.

The classical computational approach to sequence alignment employs dynamic programming algorithms, most notably the Needleman-Wunsch algorithm for global alignment (Needleman and Wunsch 1970) and the Smith-Waterman algorithm for local alignment (Smith and Waterman 1981). These algorithms guarantee optimal alignments under a given scoring scheme but operate with O(mn) time complexity, where m and n are the lengths of the two sequences being compared. When searching a query of length m against a database of total length N, exhaustive application of dynamic programming requires O(mN) operations. For modern metagenomic databases where N can exceed $10^{11}$ bases and query sets may contain millions of spacers, this quadratic scaling becomes computationally prohibitive.

Exhaustive methods that guarantee perfect recall within specified thresholds do exist for specific use cases. Tools like Sassy employ bit-parallel algorithms based on Myers’ algorithm (Myers 1999) to achieve exhaustive approximate string matching with arbitrary edit distance thresholds, while indelfree.sh (in bruteforce mode) provides exhaustive hamming distance matching. These approaches are valuable for validation and ground truth establishment on small datasets, but their computational costs scale poorly.

### Heuristic algorithms and their goal-driven design

To achieve practical performance on large datasets, virtually all widely-used sequence alignment tools employ heuristic algorithms that sacrifice guaranteed completeness for dramatic improvements in speed. These heuristics are fundamentally goal-driven: they are often designed and optimized for specific biological questions and use cases, with algorithmic choices reflecting assumptions about the expected characteristics of both queries and references.

Heuristic sequence search tools typically employ multi-stage filtering architectures. BLAST (Altschul *et al.* 1990) uses a seed-and-extend strategy: it identifies short exact matches (“seeds” or “words”) between query and database sequences, then extends these seeds using gapped alignment only in promising regions. The seed length, extension threshold, and statistical framework (*E*-values based on extreme value distribution) are all calibrated for detecting homologs across diverse sequence databases. Modern short-read mappers use similar principles but with different optimizations: Bowtie1 employs FM-index data structures enabling efficient exact substring matching followed by backtracking to allow mismatches (Langmead *et al.* 2009); Bowtie2 extends this with a multiseed heuristic and affine gap penalties (Langmead and Salzberg 2012); Minimap2 uses minimizer-based sparse seeding combined with chaining algorithms to handle long reads with higher error rates (Li 2018); StrobeAlign employs randstrobes (hash-based linked k-mers) to improve seed specificity (Sahlin 2022). Tools designed for large-scale homology searches like MMseqs2 use cascaded k-mer filtering: sequences must share sufficient k-mer matches to pass initial filtering before undergoing more expensive alignment (Steinegger and Söding 2017).

Critically, these heuristics introduce reporting biases and completeness limitations. Furthermore, many tools employ early termination strategies, reporting only the top matches or the first matches passing a threshold, which can lead to missing equally valid alternative alignments. Smart seed selection may penalize high-frequency k-mers to reduce computational burden from repetitive regions, potentially reducing sensitivity for highly abundant targets. Some tools may assume references derive from single-source assemblies and optimize for unique best-hit assignments. These design choices, while appropriate for the tools’ intended applications, currently have unknown impact in the context of spacer-protospacer matching, where queries are short (typically within 25–65 bp), searched across diverse reference sequences often comprised of multiple potential hosts genomes and mobile genetic elements (which may share genes).

### Distance metrics and their biological interpretation

Tools differ fundamentally in how they measure sequence similarity, employing different distance metrics that reflect distinct evolutionary models. Hamming distance counts only substitutions and requires sequences of equal length, making it appropriate for scenarios where length-changing mutations are rare or highly deleterious. Edit distance (Levenshtein distance) allows insertions and deletions in addition to substitutions. Affine gap distance extends edit distance by assigning different penalties to gap opening and gap extension, better modeling the biological reality.

Importantly, when comparing tools using different distance metrics with the same numeric threshold (e.g. “≤3 mismatches”), edit/gap-affine-based algorithms will naturally report more matches than hamming-based ones because they solve a more permissive computational problem. This reflects different definitions of sequence similarity rather than differences in tool quality.

### Distance metric choice and experimental evidence

For CRISPR spacer-protospacer matching in natural systems, the choice between hamming distance and edit distance has both biological and computational implications. This benchmark addresses spacer-protospacer matching in the context of inferring historical phage–host interactions in natural prokaryotic populations, which differs fundamentally from predicting CRISPR off-target effects in gene editing applications. In natural systems, we aim to identify evolutionary relationships since protospacer acquisition, where sequence divergence reflects selective pressure on MGEs to mutate and “escape” host defenses. For gene editing applications, even partial base-pairing (including alignments with indels) can cause unwanted off-target cleavage, necessitating more permissive distance metrics. Similarly, when working with low-accuracy sequencing data [e.g. Oxford Nanopore R9 chemistry (Jain *et al.* 2016)] or analyzing raw reads rather than assembled contigs (made from sufficient sequencing depth), some tolerance for indels may be necessary to account for sequencing errors, as the alignment quality cannot exceed the underlying data quality.

Experimental studies consistently report that phage escape mutations from CRISPR immunity are predominantly single nucleotide substitutions, particularly in the PAM-proximal “seed” region where mismatches have the strongest effect on targeting. Foundational work by Deveau *et al.* (2008) demonstrated that phages escape CRISPR immunity in *Streptococcus thermophilus* through point mutations in protospacers. Semenova *et al.* (2011) established that in *E. coli* type I-E CRISPR-Cas system, a seven-nucleotide seed region immediately following the PAM is critical for targeting, with mutations in this seed region abolishing immunity by reducing crRNA-guided Cascade complex binding affinity. Fineran *et al.* (2014) further showed that phages readily escape through point mutations in the PAM or seed region. More recently, Schelling *et al.* (2023) demonstrated that phage escape occurs mainly through mutations in PAM and seed regions, with preexisting mismatches at any target location accelerating emergence of mutant phages. Across these experimental systems, escape mutations are consistently reported as single nucleotide polymorphisms rather than indels. Bacterial (the host) mutation rates typically have indels occurring approximately 10× (Lee *et al.* 2012) less frequently than substitutions [albeit some extreme outliers have been reported such as ∼3× less likely (26% of total mutations) in *Acidobacterium capsulatum* (Kucukyildirim, Miller, and Lynch 2021)], and this trend is expected to be similar or more pronounced in phage genomes. Similar to their host, phage transcriptional units often contain several genes with small intragenic spaces (Hatfull and Hendrix 2011), often resulting in a particularly coding-dense genome with coding genes occupying most of the genomes (e.g. as demonstrated by Ha and Denver (2018) for multiple diverse phage families, coding sequences occupy on average >=92.4% of the entire genome). The lower indel rates align with the fact that while frameshift-inducing indels are particularly deleterious, substitutions may affect only a single amino acid residue. Frame-preserving indels (multiples of 3 bp) are extremely rare but, when observed, may be particularly strong indicators of selection. We acknowledge that most existing literature focuses on substitutions, potentially stemming from substitutions being easier to detect and characterize than indels, or a potential “assumption of expected” bias where the lack of reports about indel escape mutations may not translate to it being a less frequent phenomena. Indeed, a systematic quantitative comparisons of mutation type frequencies across diverse phage-host systems remain lacking. The only report of a verified indel escape mutation we were able to find is from a study by Paez-Espino *et al.* (2015). In that long-term coevolution experiment with *S. thermophilus* phage 2972, the authors note in the methods section “Finally, postassembly as well as comparative analyses were performed to identify SNPs, indels, and recombination events”, however indels (or gaps) are not mentioned in the main text discussing escape mechanisms, and only a single indel event is listed in the supplemental “Table S6. Phage 2972 targeting” among the escape mutations identified, suggesting even this relatively large experimental setup is not adequate to observe enough varied mutations required for statistical analysis. Of note, the authors report another type of escape mutation—large genomic rearrangements and recombination events. Viral genomes are considered highly mosaic, where genes are commonly exchanged (Hatfull and Hendrix 2011, Kupczok *et al.* 2018). While such rearrangement escape mutation are particularly interesting, we argue that in the context of sequence search tools, these would not be detectable under either hamming nor edit distance metrics, as the original biologically targeted region is either discarded (replaced by protein of similar function, but not necessarily similar nucleic sequence), or split and repositioned in different loci (in the case of internal rearrangement, such as the original spacer targeted the edge between two subsequent genes that underwent synteny altering rearrangement).

### False positives in sequence similarity searches

A critical consideration in sequence similarity searches is the expected rate of spurious matches arising by chance rather than true biological relationships. Traditionally, false positives in sequence similarity searches are considered as matches that appear similar by standard alignment metrics but arise from convergent evolution, random sequence similarity, or compositional biases rather than common ancestry or functional relationships (note: in our “Methods and Results” sections we define false positives very differently, as we lack ground truth for evolutionary relationships; see “Methods” section).

Most sequence search tools employ statistical frameworks to estimate false positive rates. BLAST calculates *E*-values representing the expected number of matches with a given score occurring by chance in a database of specified size, based on extreme value distribution theory (Altschul *et al.* 1990). Low-complexity filtering tools like DUST (Morgulis *et al.* 2006) and tantan (Frith 2011) attempt to mask repetitive regions that contribute disproportionately to spurious matches. However, determining what constitutes a “true” versus “false” positive is particularly challenging in the absence of ground truth. For short sequences (such as CRISPR spacers), this is further complicated: firstly, less positions in the alignment imply less information (e.g. the difference in likelihood as more positions are similar in both subject and query), and secondly, as the space of possible matches is very large even under low distance thresholds, and these are often searched in large genomic databases.

### Computational resource considerations

Another important consideration is computational resource requirements. Memory, storage, and availability of CPU cores are factors differing between tools. Parameter choice may also impact these factors considerably, with certain tools offering tunable parameters to trade-off between sensitivity and computational efficiency. In recent years, both spacer and viral sequence databases have been growing rapidly, with routine fold increases in size (Shmakov *et al.* 2017, Dion *et al.* 2021, Camargo *et al.* 2023). Most tools require more resources as the size of the database grows, and as this trend continues, certain workflows and tools may become prohibitively expensive to run in a reasonable time.

## Methods

### Tool selection

We evaluated several widely-used sequence alignment and search tools, spanning different algorithmic approaches and computational strategies (Table 1 and Supplementary Table S1). The tools were selected based on their availability, historical use in sequence analysis, and diversity of algorithmic approaches. The selection includes both exhaustive methods (Sassy and indelfree.sh in bruteforce mode) that guarantee finding all matches within specified distance thresholds, and heuristic methods that use various optimizations for improved speed.

**Table 1:** Evaluated tools and their characteristics.

Tool	**Indexed**	**Algorithm**	**Heuristic/Exhaustive**	**Reporting threshold**	**Year**	**Original Purpose/intended use**	**Notes**	**Key tool configuration**
Bowtie1	Yes	FM-Index (BWT)	Heuristic (backtracking)	Hamming	2009	Short read mapping	Optimized for 25–50 bp reads (max 1kbp); ungapped alignment only; backtracking heuristic limits to 3 mismatches	'—seedmms 3 -f—all -v 3'
Bowtie2	Yes	FM-Index (BWT)	Heuristic (multiseed + extend)	Affine/Edit	2012	Read mapping (w/gapped)	Uses FM-index for seeding with SIMD-accelerated DP extension; supports gapped, local, and end-to-end alignment	'-N 1 -L 15—all—very-sensitive'
indelfree.sh	No	Multi-kmer matching	Bruteforce mode is exhaustive, while in non “Indexed” mode this can be limited by selected kmer length, query length, and number of substitutions.	Hamming	introduced to bbtools Sept. 2025	Read mapping	BBTools suite is Java based, and will use available memory—so the peak memory reported herein (sourced from SLURM logs) does not equate with “minimal required memory”	Indexed mode: 'k = 8' Bruteforce: 'simd'
StrobeAlign	Yes	Randstrobes	Heuristic (syncmer thinning)	None, see, Issue 462	2022	Read mapping	Uses hash-based linked strobes (randstrobes) with multi-context seeds (MCS) for hierarchical search	'-k 15 -N 10000 -M 200'
BLAST+	Optional	Seed-Hit-and-extend	Heuristic	E-value, bit score, alignment values (%ID, coverage etc)	2009	Sequence search	BLASTN-short mode uses 7-mer seeds; reports matches based on e-value (expected hits by chance given DB size)	'-max_target_seqs 1000000 -perc_identity 84 -qcov_hsp_perc 80 -dust no -ungapped -task blastn-short'
MMseqs2	Optional	K-mer prefiltering	Heuristic (3-stage cascade)	E-value, bit score, alignment values (%ID, coverage etc)	2017	Sequence search	Double k-mer matching → vectorized ungapped → gapped SW; optimized for many-against-many searches	'—min-seq-id 0.85—min-aln-len 17'
Sassy	No	Uses a bit-parallel algorithm based on Myers’ bitpacking to perform exhaustive approximate string matching (ASM)	Exhaustive	Edit	2025	Versatile pattern matching, suggested for use in CRISPR (gene-editing) off target detection and raw-read alignments	Guarantees perfect recall; explores full edit distance landscape; supports arbitrary distances; high computational cost, uses SIMD instructions (AVX2 and NEON) when available, which most modern CPUs support	
X-mapper	Yes	Gapped x-mer pyramid	Heuristic (dynamic seeds)	Edit	2024	Read mapping	Preprint—Dynamic-length gapped x-mers with “pyramid walking” to optimize seed specificity	'—max-penalty 0.2—max-penalty-span 2—snp-penalty 1—new-indel-penalty 1.5—extend-indel-penalty 0.5'
mummer4	Optional	48-bit suffix array	Heuristic (MUM-based)	Edit	2018	Genome alignment	Identifies Maximal Unique Matches (MUMs) using enhanced suffix arrays.	'—maxmatch—nosimplify—batch = 10 000 000'

It is important to note that most of these tools were not specifically designed for CRISPR spacer-protospacer matching, but rather for more common sequence search tasks (MMseqs2, BLASTn-short), alignment/mapping of short reads to reference genomes (Bowtie1, Bowtie2, MUMmer4, StrobeAlign, X-mapper), or versatile pattern matching (Sassy, indelfree.sh). Our focus is specifically on spacer-to-protospacer sequence matching as a bioinformatics task, and we did not evaluate integrated host-prediction tools like SpacePHARER (Zhang *et al.* 2021) or iPHoP (Roux *et al.* 2023) (that perform spacer-matching internally) which rely on additional analyses such as phylogenetic evaluation or LCA determination from multiple spacer-protospacer matches.

In this work, our goal is to benchmark the different tools’ results when they are executed with suitable parameters and settings (often CLI arguments) for the task of spacer-protospacer matching. From that perspective, all tools were configured to maximize sensitivity specifically for short (spacer-sized) queries, based on available documentation. The exact commands and tool versions are provided in Supplementary Table S1. Of note, for certain tools we selected parameters based on historical use in this context (such as blastn via -task short and -ungapped) with reporting arguments designed to capture at least the range of alignments within hamming distance ≤3. For example, blastn and mmseqs search do not have explicit argument for limiting to a distance metric and value, but this can be approximated by setting a minimal alignment length (or query coverage) together with minimal identity.

### Data generation and acquisition

We evaluated tool performance using three complementary approaches with varying levels of ground truth information. The sequence statistics for the datasets (spacers and contigs) are detailed in Supplementary Table S4, and the simulation/job-generation setup is summarized in Supplementary Table S5.

### Real datasets

To evaluate tool performance in real-world scenarios, we used predicted viral contigs and CRISPR spacers from recent comprehensive databases.

*Viral contigs:* We used the IMG/VR4 v1.1 high-confidence viral contigs (Camargo *et al.* 2023), one of the most comprehensive databases of uncultured phage and viral genomes. These sequences are predicted primarily via geNomad (Camargo *et al.* 2024) scans of metagenomic data, supplemented with sequences from NCBI’s RefSeq and GenBank databases.

To focus on prokaryotic phages and exclude eukaryotic viruses (which typically lack CRISPR systems in their hosts), we applied taxonomic filtering based on ICTV classifications. Specifically, we removed contigs classified into eukaryotic virus families, orders, and classes, including but not limited to major groups such as *Adenoviridae*, *Herpesviridae*, *Poxviridae*, *Coronaviridae* (families); *Herpesvirales*, *Picornavirales*, *Bunyavirales* (orders); and *Megaviricetes*, *Alsuviricetes*, *Pokkesviricetes* (classes). After these filtering steps (starting from 5 457 198 high-confidence contigs), the final dataset contains 5 115 894 prokaryotic viral contigs with a total size of ∼79 Gbp (range: 1001–2 473 870 bp, median: 7664 bp, GC%: 44.45%). See Supplementary Table S2 for detailed statistics on the contig dataset and filtering steps.

For benchmarking, we then selected a high-quality (herein “HQ”, by filtering to keep only “High-quality” or “Reference” in the IMG/VR4 metadata field “miuvig_quality”) subset of 421 431 contigs (∼18.9 Gbp) using stratified sampling to maintain taxonomic class label distributions while focusing on reliable viral sequences (i.e. expected to be of prokaryotic virus origin). This HQ subset served as the base set for subsampling experiments and derived performance analyses. This set is available in the Zenodo deposit of this project (Neri *et al.* 2026).

*CRISPR spacers:* We used the curated spacer dataset from iPHoP (June 2025 release) (Roux *et al.* 2023), which combines CRISPR spacers from both reference genomes and metagenomes. To our knowledge, this dataset represents the largest curated spacers extracted from assembled data and is used in existing host-prediction tools. The raw iPHoP set contains 3 882 812 unique spacers (length range: 25–40 bp, median: 34 bp, GC%: 47.6%) compiled from CRISPR arrays identified primarily via piler-cr (Edgar 2007) and CRT (Bland *et al.* 2007). We applied additional filtering to remove 55 833 spacers (∼1.4% of all) with low sequence complexity or ambiguity (see below “Complexity filtering”). The entire final set of 3 826 979 spacers was used in all benchmarking analyses pertaining to the “real data”. See Supplementary Table S3 and Fig. S5 for detailed statistics on the spacer dataset composition and feature distribution.

*Complexity filtering:* In this work, our goal is to benchmark the different tools’ results given realistic input. Investigating the effects of different complexity filtering algorithms and implementations is outside the scope of this project. The different tools evaluated handle complexity and ambiguity differently—some have internal, hard-coded restrictions [e.g. blastn does not select seeds from regions with ambiguous (N) bases, but allows extending over them from another seed], or provide option to disable complexity filtering (such as -dust no in blastn). Some tools (like sassy) may allow all query sequences to contain Ns, but may allow restricting the target sequence to regions with a maximal fraction of N positions. Previous uses of blastn for this task (such as in CRISPRTarget) tend to explicitly disable complexity filtering. Some host-assignment tools (such as iPHoP) employ complexity filtering post-hoc (after collecting the search/alignment tool results). To provide a uniform starting position for all tools, so that the complexity handling is not a confounding factor, we applied a basic complexity filter to remove spacers with low sequence complexity or high ambiguity. We note that these sequences are likely not particularly informative from a biological perspective and may arise from incorrect CRISPR array prediction or extraction, and in some in-house tests for this project, we observed these disproportionately contribute to the computational resource issues associated with a non-informative matches (such as extremely massive output files detailing “potential” alignments to regions of Ns). The steps and code to reproduce the filtering are available in the project repository (spacer_inspection.ipynb). Briefly, we first calculated the fraction of each nucleotide (A, T, G, C, N) in each spacer sequence, as well as the GC%, Shannon entropy value, and the number of non-unique 6-mers (i.e. 6-mers that occur more than once in the spacer). We then filtered out spacers with any of the following characteristics: any ambiguous bases (N fraction > 0), low sequence complexity (Shannon entropy ≤ 1), high homopolymer content (any of A, T, G, C fraction ≥ 0.95), or low k-mer diversity (≥4 non-unique 6-mers). This filtering removed 55 833 spacers (∼1.4% of all) and resulted in a final set of 3 826 979 spacers used in all benchmarking analyses pertaining to the “real data”. For the exact sequence feature statistics of the spacers (mean GC%/length etc) see Supplementary Tables S3 and S4.

*Stratified Subsampling Strategy:* For benchmarking purposes, we selected an initial high-quality (HQ), representative subset of 421 431 contigs from the raw IMG/VR4 dataset as described above, termed “fraction_1”. In this benchmark, we measure the tool results on subsamples of this set for three reasons: first, we can only include the exhaustive tools (Sassy, indelfree.sh bruteforce) on the smaller fraction as they are computationally expensive, secondly by comparing the fraction to fraction variation in each tool’s result, we can estimate the tools performance consistency, and thirdly, we can investigate the effect of the dataset (fraction) size on the tools’ resource usage (CPU time, memory). To create these subsamples, we employed a “Representative Sampling” aimed at ensuring the samples reflect the entire sequence population characteristics and diversity. Specifically, each sampled subset had to include representatives from each taxonomic class, at the same proportional quantities the classes had in the 421k contig set (“HQ”). We note that this is a crucial step as the majority of the prokaryotic viruses in IMG/VR4 belong to a handful of classes causing random sampling with small sizes to have few or no representative for the various other viral lineages. Using the stratified sampling method, we created subsets of several different fractions: 0.0005 (279 contigs, 7.04 Mbp), 0.001 (421 contigs, 9.75 Mbp), 0.005 (2107 contigs, 57.06 Mbp), 0.01 (4214 contigs, 123.67 Mbp), 0.05 (21 071 contigs, 715.07 Mbp), 0.1 (42 143 contigs, 1.50 Gbp), and 1.0 (full HQ set: 421 431 contigs, 18.87 Gbp). The same set of 3 826 979 spacers was used for all subsamples and the full HQ set. Even for the smaller subsamples (0.0005–0.01 fractions), exhaustive tools did not complete within reasonable CPU time budgets (namely, 72 wall hours given 64 cpus). For this manuscript, we only include the alignments reported by any tool if that tool finished within the same time limit. A singular exception is blastn for the fraction_1 set, which was allowed to run to completion, as it is the main point of reference with regards to historical use of (any) tool for this task.

### Synthetic dataset generation

To examine each tool’s performance across diverse spacer-to-target matching scenarios, we developed a Rust-based simulation framework accessible through a Python CLI interface with fine-grained control over sequence characteristics. The simulator records the ground truth of all planned spacer occurrences, enabling differentiation between true positives (planned matches) and non-planned matches (validated alignments occurring in unplanned regions but meeting distance thresholds).

*Customizable Sequence Characteristics:* The simulation framework provides several parameters to enable more realistic sequences. Users can specify nucleotide base composition independently for spacers and contigs through either GC content percentages or explicit base frequency parameters (A, T, C, G fractions). Additional parameters control contig and spacer length distributions (uniform or normal), the range of substitution mismatches to introduce when “injecting” a spacer into simulated contigs, the number of times each spacer would be “injected” into a simulated contigs (as a range), optional indel mutations (insertion and deletion ranges), and the proportion of spacers to reverse complement. Additionally, a semi-synthetic option is permitted—where either an external (existing) spacer or/and contig set is provided by the user.

*Sequence Generation Process:* The Rust-based core of the simulator generates DNA sequences using weighted random sampling from the nucleotide alphabet (A, T, C, G) based on the specified base composition. For each position in a sequence, a nucleotide is selected with probability proportional to its configured frequency. Sequence lengths are sampled from the specified distribution type: uniform distributions select lengths with equal probability across the range; normal distributions sample from a Gaussian with mean at the midpoint and standard deviation chosen to span the range; and bell curve distributions use a Beta distribution to create length variation with mode near the center. Before actual sequence modifications begin, the simulator creates an “injection” plan (“injection” refers to replacing a contiguous region of a contig with a spacer sequence, creating what we refer to as “planned spacer occurrences”—this should not be confused with “insertion” indel mutations, which add bases within sequences). This plan predetermines which spacers will be placed into which contigs, how many times each spacer appears, which occurrences will be reverse-complemented, and what mutations each will receive. The plans is then executed across separate processing threads, so that the load (number of spacers and to generate contigs) is balanced by total spacer-placement lengths so that a similar value is assigned to each thread. To prevent individual contigs from becoming overly saturated with spacers, the simulator calculates contig utilization—the percentage of total contig base pairs that will be occupied by injected spacers—and reports this value (note—in the simulation runs described below, this value remained mostly below 2%). During injection, each spacer replaces an existing contig region of equal length at a randomly selected position, thereby preserving the original contig length while creating the “ground truth” alignments. When injecting spacers into these predetermined positions, the simulator applies mutations in a defined order: first, indels (insertions and deletions) mutations are applied separately at random positions (note—this option was not used in the current project); second, substitutions (“mismatches”) replace bases with different nucleotides (preventing identity-preserving substitutions like A→A) at random positions. The number of substitutions, the injected spacer coordinates on the contig, the strand, and the contig and spacer identifiers are recorded as the (planned) ground truth. The final outputs the contigs and spacer sequences (as FASTA files), and the ground truth (in tabular format).

*Comparison to real spacers:* Rather than using purely random sequences and uniform distributions, for the sets described here, we configured the synthetic data generation to match certain characteristics of the real datasets, namely the filtered iPHoP spacer set and the HQ IMG/VR4 dataset noted above as “fraction_1”. Specifically, we set the GC content to approximately 49% (spacers), and 46% (contigs), and configured contig lengths to be selected under normal distribution from a realistic range (mostly, 1501–200 000 bp, see Supplementary Table S4 for complete parameters). To illustrate the ability of the simulated sequences to mimic the real data sets, we calculated and compared several features (e.g. k-mer repeatability, entropy, base frequencies etc; see Supplementary Fig. S4 and notebooks: spacer_inspection.ipynb). Most analysed features indeed appear similar for the simulated and real sequences, with the exception of that in some complexity measures (e.g. k-mer repeatability), the real spacers have a slightly wider range of value, suggesting a minor amount of the real spacers have more extreme values in this regard.

*Simulation Dataset Variants:* We generated multiple synthetic datasets with varying sizes and characteristics to evaluate tool performance under different conditions and to assess the tools consistency (for similar reasons as the real-data subsamples). The datasets follow a naming convention ns_[n_spacers]*nc*[n_contigs] indicating spacer and contig counts. Smaller datasets (ns_50000_nc_5000, ns_75000_nc_5000, ns_75000_nc_10 000) used 25–40 bp spacers, 10 000–150 000 bp contigs under normal distribution, 1–5 spacer insertions (placements, injections) per each simulated spacer, and 0–5 substitution mismatches, with all tools evaluated at hamming distance ≤5. Medium-sized datasets (ns_100000_nc_10 000, ns_100000_nc_20000) used similar parameters with all tools at hamming distance ≤5, while the largest dataset (ns_500000_nc_100000 with 500k spacers and 100k contigs spanning 10 000–550 000 bp) was limited to hamming distance ≤3 and excluded exhaustive tools (Sassy, indelfree bruteforce) due to computational constraints. Additionally, we created a specialized high-insertion-rate dataset (ns_500_nc_5000_HIGH_INSERTION_RATE) with only 500 spacers but 100–2500 injections per simulated spacer to test tool behavior under extreme multi-mapping scenarios, and a minimal dataset (ns_100_nc_50000) with 100 spacers across 50 000 contigs (2500–850 000 bp range, 1–3 injections, 25–45 bp spacers) for rapid validation. All synthetic datasets configured spacers and contigs with GC content matching real data (49% and 46% respectively), used normal length distributions, and included 50% reverse-complemented injections to reflect biological reality. See Supplementary Table S4 for complete simulation parameters and resulting dataset statistics. Note, not all tools were able to complete all runs within the same time limit for all subsamples (see “Computational Resource and Runtime Tracking” for details).

### Semi-synthetic dataset

This set uses the existing simulation framework, but instead of generating both spacers and contigs from random sequences, we use the same filtered spacer set as the real-datasets, and generate synthetic contigs matching the sequence characteristics of the HQ IMG/VR4 dataset (fraction_1, see Supplementary Table S2 for dataset details). Note, the spacers are not “injected” into the simulated contigs (using the –number-spacer-insertions 0 0 option of the simulate command). We primarily use this set to estimate “non-planned” match rates in a realistic spacer set sequence composition context, and realistic search space context. In this context (0 planned spacer occurrences) we expect all identified matches to reflect chance similarity. We note that this set is considered “large” (by design, similarly to the fraction_1), hence we are only able to use an aggregate of the verified non-exhaustive tools, and only under hamming distance ≤3. This suggests that the actual count and rate may actually be larger. See the “Non-planned Match Rate Estimation” section below for details on the definition of “non-planned” matches.

### Coordinate tolerance and unique region counting

When aggregating results across tools, we implement coordinate tolerance matching to handle slight boundary differences in reported alignments. We observed tools may report alignments with minor variations in start/end coordinates (typically 1–5 bp) due to different handling of terminal mismatches or gaps (see Supplementary Note 1 for detailed example). We use a default 5 bp tolerance when merging alignments to count unique spacer-contig regions. This approach reduces double-counting of essentially identical matches, accounts for valid algorithmic differences in gap versus substitution placement at alignment boundaries (or variation in tool-specific “clipping” behavior), and ultimately enables a fair comparison of tool results coverage (total unique regions detected). All reported alignments from all such regions are verified separately, by extracting the reference contig region and realigning to the spacer sequence (see “Alignment verification and distance metric calculation” section below).

### Alignment verification and distance metric calculation

For comparing alignments across tools, we use hamming distance (counting only substitutions) as our primary distance metric, with biological and computational justification provided below. Our benchmarking CLI tool supports setting three distance metrics: (minimal) hamming distance, (minimal) edit distance, and gap-affine (by measuring the edit distance from an alignment with a user provided cost matrix and gap penalties). However, for the analyses presented here, we focus primarily on hamming and edit distances ≤3 for most datasets, with (only) hamming distance of up to 5 substitutions used in datasets where computational resources permitted. As noted in the introduction, we recommend prioritizing hamming distance for spacer-protospacer matching in the context of phage–host interactions, as this better reflects the predominant mutation types observed in experimental studies of phage escape from CRISPR immunity. Meanwhile, in the context of gene-editing, a minimal edit distance might serve as a more appropriate metric for off-target effect prediction. Despite this recommendation, we have attempted to compare hamming and edit distance effects empirically in our analyses (when computationally feasible). We note that while in practice, we observed near-complete agreement between the minimal edit and gap-affine distance metrics under the conditions tested, these are not identical measurements—a gap-affine distance metric allows for more flexible gap placement and scoring, aimed at capturing biologically relevant (i.e. sharing a common ancestor) relationships, while the minimal edit distance metric will prioritise the minimal set of edits (substitutions and indels) regardless of their evolutionary likelihood (e.g. the higher rarity of indels compared to substitutions).

*Distance Verification Methodology:* To ensure consistent and accurate distance calculation across tools that use different internal alignment algorithms and scoring schemes, we independently recalculated distances for all reported alignments post-hoc. This is done separately for the different distance metrics: for the minimal edit distance metric, the python version of the edlib library (Šošić and Šikić 2017) is used (edlib.align) to align the sequences, and all insertions, deletions, and substitutions are counted. For the hamming distance metric, we measure the number of non-identical residues in the best (ungapped) aligned region, specifically: we slide the sequences across each other to the point of minimal distance, and consider all spacer bases outside the alignment as clipped (unaligned), and add them to the count of substitutions. This is done as hamming distance by definition can only be measured by strings of identical length, and as not all tools tested perform and report an ungapped alignment. The CLI tool we provide includes a third option—the edit distance under a gap-affine alignment, via parasail’s (Daily 2016) implementation of the Needleman-Wunsch global alignment algorithm, supporting user supplied gap opening and extension costs. We note that the gap-affine based comparisons are not presented in this current version.

### Performance definitions and calculation

We defined the ground truth and classified alignments slightly different for synthetic and real datasets, as for the larger real-data sets we do not have the results of an exhaustive tool to establish a complete set of alignment.

Specifically, for synthetic datasets, we define three categories: (i) **positive_in_plan:** Alignments matching planned spacer occurrence coordinates (±5 bp tolerance, see Coordinate Tolerance and Unique Region Counting). These represent the intended ground truth matches, i.e. sequence alignments consistent with the simulation pre-planned design at known coordinates, strands, and given distance from the raw (query-version) of the spacer. (ii) **positive_not_in_plan:** Alignments reported within the allowed distance threshold passing our independent validation, but occurring outside planned regions. These represent chance similarities at the specified distance—not false positives in the technical sense (these are valid alignments), but non-planned matches that may indicate increased background noise under certain conditions (larger distance threshold, larger search space, see the “Non-planned Match Rate Estimation” section below). (iii) **invalid_alignment:** Alignments that fail the independent alignment verification, or exceed quality thresholds. These are “true” (in the classical sense) false positives, and we note these tend to result from tool-specific reporting artifacts: not all tools support limiting reported alignments within a specific distance metric and threshold (e.g. strobealign does not have any explicit score based option to control what alignments are reported), and the parameters we provide to these tools can only approximate it (such as minimal identity or minimal query coverage). Note that the parameter choice is meant to include at-least all matches within our analysis scope and recommendation (hamming ≤3) but often includes a larger range. In the context of this project, we discard these “invalid-alignments” and do not investigate them further.

We extend these definitions to the real datasets by treating all (validated) alignments reported by all tools as “positive_not_in_plan”.

In this project, we define a tool’s “Recall” as the positive rate, or fraction of positives detected by the tool. When this fraction is calculated out of the planned alignments only (not including the non-planned), we specify it by noting “non augmented” (e.g. “non-augmented recall”). This reflects our choice to consider the non-planned validated matches as positives. We argue that from a pure sequence alignment perspective, they are as correct (within the distance threshold) as the planned, and that for the real datasets, where we do not have a complete set of planned matches, this is the only way to calculate recall in practice. Furthermore, we specifically recommend the distance metric and threshold choice (hamming ≤3) as we estimate to be considerably lower than the number of real alignments we observe in the real data-set (this is not the case for higher distance thresholds or for different distance metrics). Additionally, we note that metrics such as precision ($\frac{\mathrm{TruePositives}}{\mathrm{TruePositives}+\mathrm{FalsePositives}}$) are not applicable in this context—as were we to define the non-planned matches as false positives (for the synthetic sets), we could artificially control this value by adjusting the simulation parameters (i.e. the number of planned spacer occurrences). We also note that in this framework “true negatives” (“all correctly not reported matches that do not actually align”) is not a sensible or useful definition. By extension we can not compute certain common performance metrics such as specificity.

We acknowledge that a limitation of this system is the lack of a complete set of all positives for datasets too large to be exhaustively searched (using sassy and indelfree.sh bruteforce mode). In such cases, the positive set is essentially the union of all valid alignments reported across all tools.

*Non-planned Match Rate Estimation:* Using the synthetic datasets allows us to estimate the frequency of these reported alignments as a dependency of distance metric and threshold, and of the search space size. As we control the exact details in the simulated runs, we generate a ground truth table, where the location, number of mismatches, spacer and contig identifiers of each planned spacer occurrence is recorded. By combining this ground truth with the different tool results (particularly the exhaustive ones), we are able to identify (and subsequently verify) any reported alignment—and record the number of valid (within distance threshold) alignment not explicitly planned (occurring in regions other than those in the simulation plan). We expect these non-planned matches to represent chance similarities arising from sequence composition and length. To estimate the rate of such non-planned matches, under a given distance threshold, we divide the number of validated non-planned matches by different representations of the total search space size: a metric reflecting both spacer and contig set sizes in bp (sum of spacer lengths * sum of contig lengths), or the product of the number of spacers and contigs (e.g. per n spacer and m contigs). Realistically, total spacer length is negligible compared to total contig length, however the product of the number of spacers and contigs assumes every contig and spacer affect the search space equally.

We quantified non-planned match rates using the exhaustive search tools under varying hamming (indelfree bruteforce) and edit (sassy) distance thresholds (1–5). For large datasets (where the use of exhaustive search tools is too computationally prohibitive), we either reduced the threshold the range (1–3) if possible. For the full set (fraction_1, the semi-synthetic set, and the largest of the simulated runs) we resorted to using the aggregation of the non exhaustive tools (namely Bowtie1 and blastn) results as proxy. See Supplementary Tables S7 and S8 for more details.

### Computational resource and runtime tracking

While the primary focus of this study was to evaluate the ability of each tool to accurately identify spacer-protospacer matches, computational resources are a limiting factor for certain dataset sizes. This is particularly relevant for the exhaustive tools (Sassy and indelfree.sh in bruteforce mode), which have high computational costs.

The CLI benchmarking tool we developed utilises hyperfine (Peter 2023) for local execution of tools (suitable for smaller datasets), and a SLURM [Simple Linux Utility for Resource Management (Jette *et al.* 2002)] based method (suitable for larger datasets ran on high-performance-compute clusters), where we captured the resource usage via SLURM’s built-in accounting system (sacct), accessed through a custom Python wrapper. For consistency, all analyses herein were performed using the SLURM tracking approach. Both approaches allow us to capture detailed resource usage metrics, including wall clock time, CPU time, and peak memory usage, which are critical for understanding the practical feasibility of each tool under different conditions. All SLURM job logs were retained for reproducibility and are available in the Zenodo repository. All tools were allocated the same CPU and memory resources (64 threads, 512 GB RAM) to ensure a fair comparison, and the same maximum wall time limit (72 hours) was applied to all runs. If a tool exceeded the wall time limit, it was terminated and marked as “timed out” for that dataset, and no results were recorded for that run. The only exception to this was blastn for the fraction_1 set, which was allowed to run to completion as it is the main point of reference with regards to historical use of (any) tool for this task.

We note that memory (RAM) tracking for some java tools (particularly BBMap suite tools such as indelfree.sh) can make it seem as if they use all available memory as the java virtual machine may not explicitly report cleared, unused memory, in a way visible to the SLURM accounting system. This means that the SLURM reported peak memory usage may not indicate the actual minimal requirement of these tools. For tools that require generating an index file (or any additional obligatory steps and commands) prior the actual search/scan/alignment command, we include the index construction time (or the additional commands) in the total runtime of a tool as these represents real computational cost, though these would represent one-time cost in case of repeated searches of the same data.

### Versioning and reproducibility

All tools were installed and managed using conda (2026) [via the mamba (Mamba 2026) package manager] in isolated environments. To prevent dependency conflicts and ensure reproducibility, most tools were installed in dedicated environments. Environment activation time was excluded from performance measurements to focus on actual tool runtime. The exact versions and configurations of all tools were recorded in environment files, allowing for exact replication of our testing environment.

All benchmarks were performed on a standardized high-performance computing node to ensure consistency. The hardware environment consisted of a dual-socket system equipped with two AMD EPYC 754 332-Core Processors, providing a total of 64 physical cores and 64 threads. The CPUs operated at a frequency of 3705.616 MHz with a 32 MB L3 cache. The system was equipped with 512 GB of RAM ($5.5\times 10^{11}$ bytes) and utilized a SAMSUNG MZ1LB1T9HALS-00007 storage unit managed via an NFS file system. The software stack was deployed on Linux (kernel version 4.18.0–553.58.1.el8_10.x86_64) using an x86_64 architecture. Benchmarking scripts were executed using Python 3.10.19.

### Extensibility

The benchmarking framework is designed to be expandable through the integration of new tools. Each tool/software configuration is saved as a separate JSON file, which includes the exact commands and conda/mamba environment it uses. This configuration files can use placeholder variables which the main benchmarking software replaces (according to the users’ CLI arguments) during execution (such as {threads}, {contigs_file}, {spacers_file}, {output_dir}, and {results_dir}). A new JSON file can be added manually or via bench.utils.tool_commands: add_tool function in a semi-automated method.

## Results

### Selection of distance metric and threshold values

To investigate the effects of metric and threshold choice on spacer-target alignments, we first wanted to evaluate how frequently short sequences such as CRISPR spacers (20–45 bp) would align to target sequences, either perfectly or allowing for some mismatches and/or gaps, and with variable sizes for the target sequence database. To that end we utilized both simulated and semi-synthetic datasets to quantify non-planned match rates (see “Methods” section). As noted in the methods (see Performance definitions and calculation), these non-planned matches are not considered “false positives” in the traditional sense, as they represent valid alignments within the distance threshold (verified independently of which tool reported them) that occur in regions not explicitly included in the simulation plan. In this section, we use these non-planned matches to estimate the expected “background noise” under different distance metrics (edit versus hamming), thresholds (at exact n distance or cumulative up to n distances), and search space sizes (based on simulation/dataset parameters). This estimation of the expected level of “noise” in spacer-target alignment results was then used to more robustly interpret results obtained from planned matches or from real data for which no ground truth is available.

To empirically assess the expected frequency of non-planned matches (valid alignments occurring in regions without planned spacer occurrences, see Performance definitions and calculation) under different distance metrics and thresholds, we analyzed both fully synthetic and semi-synthetic datasets. In this context the non-planned (validated) matches are considered as genuine sequence similarities arising from chance. We quantified non-planned match frequency using three metrics: (i) **Absolute count**: total number of verified non-planned alignments. (ii) **Per spacer per Gbp target**: non-planned count divided by the number of spacers and the total contig length in Gbp ($\frac{\mathrm{count}}{n_{\mathrm{spacers}}\times \mathrm{conti}g_{\mathrm{bp}}/10^{9}}$). This metric normalizes for dataset scale differences. (iii) **Per Gbp^2^ search space**: non-planned count divided by the product of total spacer length and total contig length in $\mathrm{Gb}p^{2}$ ($\frac{\mathrm{count}}{\mathrm{space}r_{\mathrm{bp}}\times \mathrm{conti}g_{\mathrm{bp}}/10^{18}}$). This metric accounts for the full combinatorial search space.

**Figure 1:**
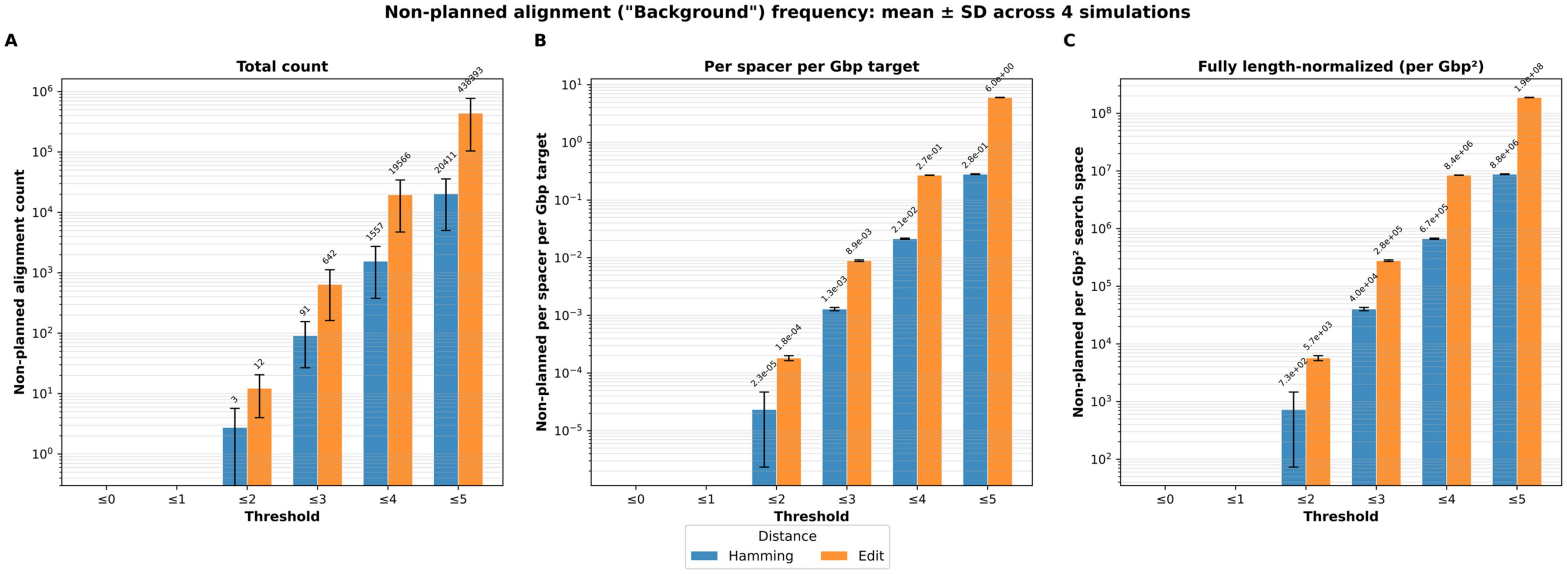
Non-planned alignment (“background”) frequency: mean ± SD across 4 fully synthetic simulations (50 000–100 000 spacers × 5000–20 000 contigs). (A) Absolute count of non-planned matches at each distance threshold (0–5). (B) Non-planned matches normalized per spacer per Gbp of target sequence. (C) Non-planned matches normalized per ${\mathrm{Gbp}}^{2}$ of total search space (spacer-bp × contig-bp). Blue bars: hamming distance (substitutions only). Orange bars: edit distance (substitutions + indels). Error bars: standard deviation across simulations. At hamming ≤3, the per Gbp^2 rate is approximately 40 000 while at edit ≤3 the rate is approximately 270 000–290 000 (a 6–8 fold increase). Full per-simulation breakdowns are provided in Figure S9.

Briefly, computational constraints limited exhaustive edit-distance search to smaller fully synthetic datasets. Using exhaustive tools (Sassy and indelfree), we observed substantially higher non-planned match rates when allowing indels (Fig. 1). At edit distance ≤3, non-planned match frequencies exceeded hamming distance ≤3 rates by approximately 5–10 fold depending on the dataset. This increase reflects both biological permissiveness and the larger combinatorial space of edit distance relative to substitutions-only hamming distance. The search-space-normalized rates were relatively consistent across simulations, while very small thresholds (0 and ≤1) noisier due to low event counts.

Similarly, we use the semi-synthetic dataset (see Semi-Synthetic dataset) to estimate non-planned match rates at a scale analogous to the “fraction_1”. We note that this dataset size was too prohibitive for the exhaustive and edit-distance based tools, hence we rely on the aggregate of the non-exhaustive tools as a proxy for the total number of non-planned matches, which is likely an underestimate. Similarly, these results only estimate the hamming-based non-planned match rates. Specifically, the total amount of non-planned matches was 1 for 0 hamming threshold (i.e. exact match), 49 for an hamming distance of ≤1, 2354 matches at ≤2 hamming distance, and 56 273 at ≤3 distance (see Table 3 for the results at hamming ≤3 compared to the fully synthetic simulation mean).

The approximately 0.5-fold lower normalized rates in the semi-synthetic dataset relative to fully synthetic simulations may stem from the real spacer sequences having compositional features (conserved motifs, non-uniform k-mer distributions, locus-specific GC variation) that alter the rate of chance similarity. Therefore, these values should be interpreted as qualitative indicators of relative (between edit vs hamming and at increasing thresholds) background alignment rather than exact predictive rates, as the simulated contigs only capture basic sequence features (namely GC% and length ranges), but not all structural features of real viral genomes.

Given this estimation of chance similarity and background noise, we selected hamming distance ≤3 as the primary threshold for most subsequent analyses, but we acknowledge that for certain specific research questions and scenarios, an edit distance may be more appropriate, and we include some specific analyses to that effect.

### Tool performance across distance thresholds

**Figure 2:**
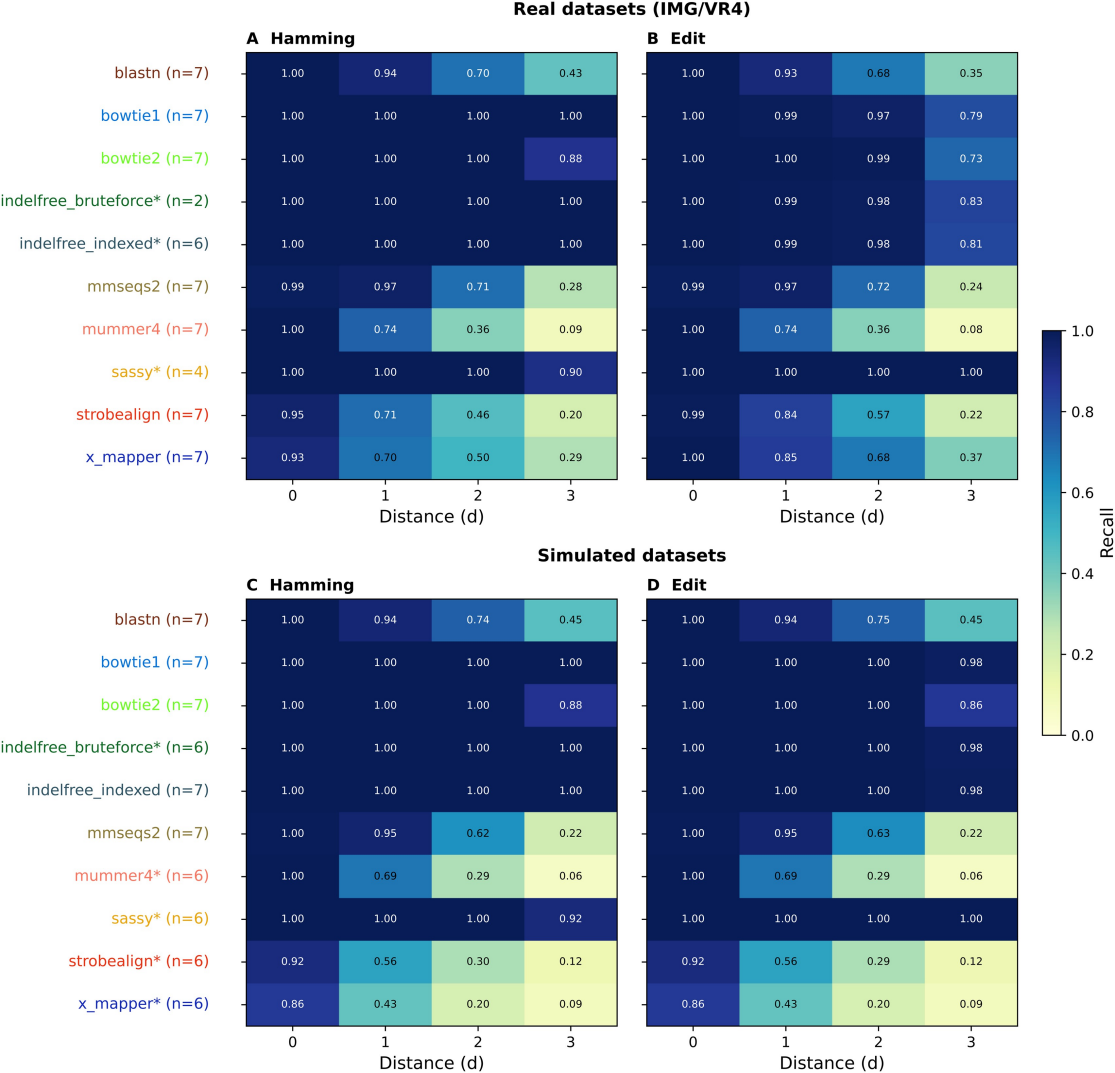
Tool recall (detection fraction of unique valid aligned regions) across distance thresholds. The upper row shows IMG/VR4 (fractions) results and bottom row shows synthetic datasets; columns compare hamming and edit distance analyses. An asterisk “*” is noted after tools that did failed or timed out, and the total number of successful runs (subsampled fraction for the IMG/VR sets, and independent measurements for the simulated set) are noted in brackets after each tool’s name, and the value in each cell is the mean of those run specific recall values. Note—the values are at exact distance (unlike at a “max” distance), i.e. regions that aligned with distance n are not considered for distance *n* + 1.

We next evaluated the ability of individual tools to recover all valid matches, summarized in Fig. 2 as the mean tool recall across a given spacer and target dataset type.

While we focus on hamming distance for the simulated datasets (i.e. in the current version, we only simulate substitutions, and not indels), we included non-hamming-based tools commonly used for sequence searching/alignment. For both dataset types, we aggregate the tool results and validate them under both hamming and edit distances separately (see methods). We note however that this naturally penalizes tools that might produce “better” alignments under their metric (especially if the tool limits the reports to the best scoring alignment per reference region), which might not align with the “best” (i.e. minimal) hamming distance (for a given region). For example, having a single gap in the middle of a spacer-target alignment could produce a better (or valid/below certain distance value) alignment under edit distance, but if the same aligned region is evaluated under hamming distance, it is likely that half of the aligned region will include multiple consecutive substitutions. Thus an important limitation here is that more or less reported alignments per-tool under **all** metrics, are not necessarily failures of the tool to detect the matches, but rather a reflection of algorithmic differences in definitions. We also note that in practice, the ability of some of the strictly-hamming based tools (Bowtie1and indelfree in indexed mode) to report high recall values under some of the measured edit-distance for the real-life datasets, suggest that under these low (<=3) distances, there is a large overlap between the actual alignable targets. The results presented in Fig. 2 should therefore be informative once a specific metric is selected.

With regards specific between-tool recall, performance varied systematically with distance threshold. At distance 0 (exact matches), multiple tools achieved recall above 0.95 on both simulated and real datasets, including all tools except strobealign and x-mapper. As mismatch tolerance increased, differences between tools became more pronounced.

Across both simulated and real datasets, Bowtie1 (finished in allocated time for all runs) and indelfree.sh (in index-mode, finished within allocated time for all simulated run, and for 6 out of 7 of the real-dataset fractions) remained the highest-recall heuristic tools at hamming distance ≤3, while tools optimized for different mapping contexts (notably StrobeAlign, MMseqs2 and MUMer4) showed larger recall losses at higher mismatch levels. In edit-distance analyses, exhaustive Sassy provided the expected upper bound when runs were computationally feasible, while heuristic edit-capable tools showed variable sensitivity depending on dataset scale and mismatch tolerance.

Performance patterns were consistent between simulated and real datasets, though the smaller simulated dataset size and availability of exhaustive tools on more synthetic data enabled more precise recall measurement. A notable exception were strobealign, x-mapper and (to a lesser degree, and in simulated runs only) mmseqs2, for which the calculated recall rates varied between individual runs (see Supplementary Fig. S8). These variations were observed in both simulated and real-dataset runs.

Complete per-tool, per-threshold recall values are provided in Supplementary Table S6. In this regard, it is important to note that within the allocated or available total CPU time per job, the largest “real-dataset” IMG/VR fraction amenable to the exhaustive tools was 1% (sassy, edit) and 0.1% (indelfree_bruteforce, hamming; see section below regarding computational resource usage for more details).

Pairwise tool comparisons measuring the set-difference of reported alignments between two tools were consistent with these recall trends (Supplementary Fig. S1), indicating the same alignments were reported by multiple tools at low distance values, and the number of tool-specific differences quickly grew with increasing distances considered.

### Computational resource requirements and scalability

**Figure 3:**
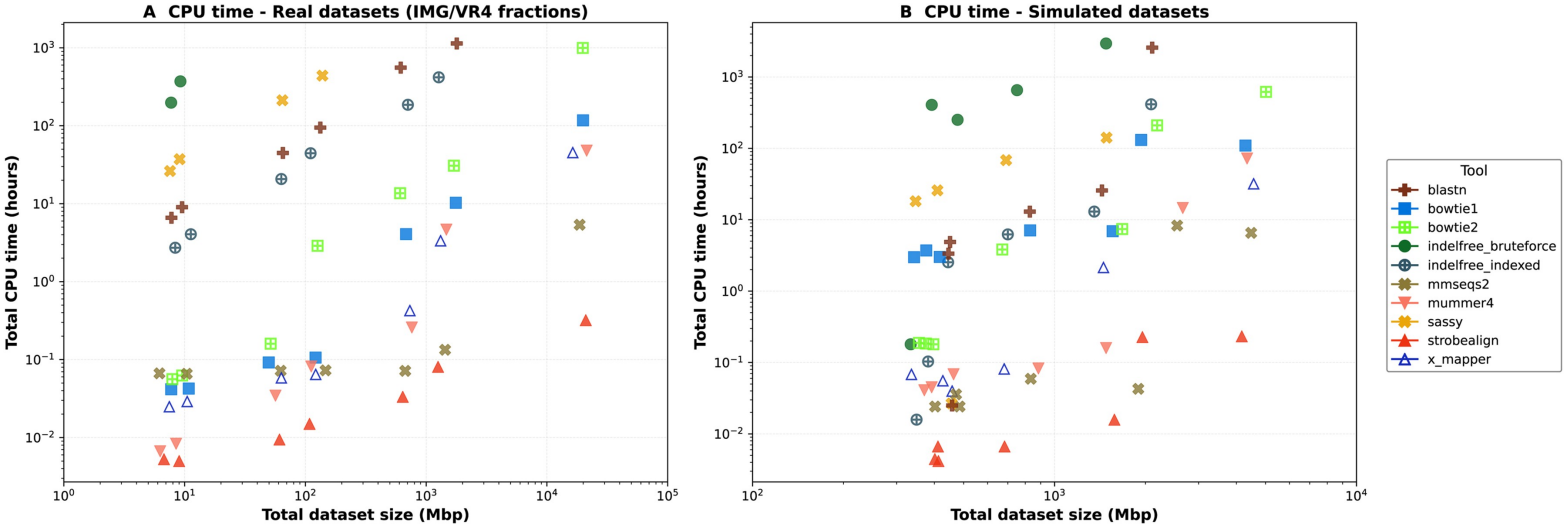
Total CPU time scaling with dataset size (log-log scale). (A) Real IMG/VR4 subsampled datasets (fractions 0.001–1.0, approximately 10 Mbp to 18.9 Gbp target sequence). (B) Simulated datasets with varying numbers of spacers and contigs (see Supplementary Table S4 for dataset details). Marker shape and color encode tool identity. Note, *x*-axis “jitter” was applied to avoid overlapping marker positions. BLASTn runtime for fraction_1 was extrapolated from partial completion (1.69M of 3.83M spacers processed within the 72 h wall time limit; see Supplementary Table S9 for all SLURM accounting results).

Computational resource requirements vary dramatically across tools, reflecting fundamental differences in algorithmic approaches and trade-offs between sensitivity and efficiency (Fig. 3). Understanding these resource requirements is essential for practical tool selection, particularly as CRISPR spacer and viral databases continue growing rapidly.

CPU-time trends in Fig. 3 show large differences in practical scalability, with exhaustive tools [Sassy (orange “x” marker) and indelfree_bruteforce (teal “□” marker)] typically requiring substantially longer runs even on smaller datasets. Missing points indicate tool-dataset combinations that did not produce a completed value for plotting (e.g. timeout, failed runs, or runs excluded from the plotted subset); the corresponding SLURM-derived records are provided in Supplementary Table S9, and the logs (stdout and stderr) for all runs are available in the Zenodo repository.

Among heuristic methods, MMseqs2, StrobeAlign showed the most favorable wall-time behavior across increasing dataset size, followed by MUMer4 and X-mapper. Bowtie1 and Bowtie2 typically had similar runtimes for the same datasets, except for the smaller simulated runs where bowtie2 required considerably less time to complete. BLASTn and indelfree.sh in indexed mode typically required substantially more time than the other tools, and neither successfully finished processing the fraction_1 (IMG/VR, real dataset) within the allocated 72-hour wall-time limit (note -BLASTn was allowed to continue until completion solely for comparison of results as it is the main tool historically used for spacer-protospacer matching).

As expected, exhaustive tools were markedly slower; notably, indelfree_bruteforce remained computationally expensive even at intermediate subsamples. Surprisingly, Sassy, despite being an exhaustive edit-distance based tool, showed more favorable runtime than indelfree_bruteforce (a strictly hamming distance based tool), reflecting that algorithmic constraints can be alleviated by dedicated implementation optimizations.

With regards to memory usage (Supplementary Fig. S7), SLURM peak values differed by up to one to two orders of magnitude among tools. This metric should be interpreted together with runtime because some tools reduce memory pressure by partitioning reference data or increasing disk I/O, which can increase wall time. Reported peak memory for Java-based tools (x-mapper and BBMap-suite tools such as indelfree.sh) may also be affected by JVM allocation behavior relative to SLURM RSS accounting.

The paired real/simulated design also helps separate different scaling effects. In simulated runs, both spacer and contig counts vary (Supplementary Table S4), while in IMG/VR4 runs the spacer set is fixed and only contig-side subsampling changes. This contrast suggests that, for some tools, peak memory is influenced more strongly by index structure and longest-contig properties than by total contig-set size alone. Complete resource values and run-level details are provided in Supplementary Table S9.

### Performance as a function of query (spacer) abundance in reference

In the fraction_1 analysis, we observed occurrence-dependent sensitivity effects in most tools, where spacers with very high occurrence in the target set showed reduced recall (Supplementary Fig. S3). We corroborated this behavior with a dedicated high-insertion simulation (ns_500_nc_5000_HIGH_INSERTION_RATE), where each spacer was inserted between 100 to 2500 times in simulated contigs (Supplementary Fig. S2). The strongest degradations were tool-specific and most were only visible at the highest occurrence bins. Importantly, ultra-high occurrence spacers (>1000 occurrences) represented a very small fraction of matched spacers (<0.01%; Supplementary Fig. S6), so this effect is measurable but limited in overall contribution to aggregate recall and likely to be limited in real-case analyses.

## Discussion

Our analysis across fully synthetic, semi-synthetic, and real IMG/VR4 datasets demonstrates that algorithmic design assumptions substantially influence spacer-protospacer detection sensitivity. The quantitative comparison of ten tools, including two exhaustive methods, enables evidence-based recommendations while revealing systematic differences rooted in the computational problems each tool was designed to solve.

### Distance metric choice and practical thresholds

The empirical non-planned match rates measured in this study provide qualitative grounds for threshold selection. In the semi-synthetic dataset (3 826 979 real spacers searched against 421 431 simulated contigs), the number of validated non-planned matches rose sharply with distance: 1 at exact match, 49 at hamming distance ≤1, 2354 at ≤2, and 56 273 at ≤3 (Table 3; Supplementary Table S8). Analyses of fully synthetic datasets using exhaustive tools further showed that edit distance ≤3 produced approximately 6–8 fold more non-planned matches than hamming distance ≤3 at the same numeric threshold, and that the number of non-planned matches quickly become overwhelming beyond distance cutoffs of ≤3 (thousands to tens of thousands in our simulations, Fig. 1; Supplementary Table S7). These quantitative observations, combined with the biological evidence reviewed in the introduction for substitution-dominant phage escape mutations (Deveau *et al.* 2008, Semenova *et al.* 2011, Fineran *et al.* 2014, Schelling *et al.* 2023), support hamming distance ≤3 as an appropriate default threshold for spacer-protospacer matching in the context of host–MGE interaction inference, in most scenarios.

The practical implications of these rates are scale-dependent. For small datasets, such as a single or few phage genomes (total target sequence well below approximately 1 Gbp) searched against spacers extracted from several host genomes, the per-search-space-normalized rates suggest that non-related matches at hamming ≤3 are infrequent. For large-scale host assignment analyses where the total search space approaches or exceeds the scale tested here, the expected number of non-planned matches should inform downstream interpretation. At such scales, post-hoc filtering—for example, requiring multiple supporting spacers per host-virus pair, incorporating phylogenetic context, or applying stricter distance thresholds—is warranted to mitigate the accumulation of chance matches. Edit distance metrics may be appropriate under narrowly defined conditions: when analyzing data from sequencing platforms with elevated indel error rates (e.g. Oxford Nanopore R9 chemistry), when explicitly characterizing escape mutation types in controlled experimental systems, or when predicting gene-editing off-targets where multiple short indels may still inform regions that could base-pair or lead to cleavage. Outside of these scenarios, the substantially higher non-planned match rates associated with larger edit distances (6–8 fold at ≤3) argue against its routine use for inferring host–MGE interactions from natural populations.

### Tool performance and algorithmic considerations

A central observation is that tools differ in recall rate not because of implementation deficiencies, but because their algorithms solve different computational problems. Edit/affine-based tools (Bowtie2, Sassy) and hamming-based tools (Bowtie1, indelfree.sh, BLASTn-ungapped) will, by definition, identify different match sets when applied at the same numeric threshold, as they operate under different definitions. This fundamental distinction must be considered when interpreting and comparing results. Within the hamming distance analyses, Bowtie1 consistently achieved the highest recall among heuristic methods across both dataset types and distance thresholds (Fig. 2). Performance patterns were notably consistent between simulated and real datasets (Supplementary Fig. S8), supporting the validity of our simulation framework and suggesting these findings generalize across database compositions.

At higher mismatch levels, differences between tools became more pronounced. Tools originally designed for mapping short reads against single-source reference assemblies (specifically StrobeAlign) showed steeper recall declines, consistent with seed-selection heuristics that penalize high-frequency k-mers or that assume reference uniqueness. Similarly, we observed occurrence-dependent sensitivity effects (see Performance as a function of query abundance), where spacers with many valid targets in the reference showed reduced detection rates in most heuristic tools. While spacers with extremely high occurrence frequency (>100 occurrences) represented a negligible amount of the total matched spacers (<0.01%) (Supplementary Fig. S6), the effect was measurable across a broader range of occurrence frequencies and may matter for analyses specifically targeting broadly conserved genomic regions.

The computational resource analysis reveals substantial differences in scalability (Fig. 3; Supplementary Fig. S7). While index construction is often a one-time upfront cost, we included it in the measured resource budget because it represents real computational expenditure. Among heuristic tools, Bowtie1 combined high recall with favorable runtime scaling, whereas BLASTn-short under sensitivity-oriented parameters exceeded the 72-hour wall-time limit on the full fraction_1 dataset. The practical feasibility of exhaustive tools depends strongly on dataset scale. For targeted analyses of specific host-phage systems or comparisons among defined lineages, where the total subject sequence is typically well below 1 Gbp and the number of query spacers ranges from tens to thousands, exhaustive tools such as Sassy and indelfree.sh bruteforce remain tractable and provide the advantage of guaranteed complete detection. In contrast, for metagenomic datasets where the combined contig set routinely exceeds 1 Gbp and spacer sets may contain millions of sequences, the computational cost of exhaustive search becomes prohibitive. For such analyses, heuristic tools—particularly Bowtie1—offer near-perfect recall at hamming distance ≤3 with orders-of-magnitude lower resource requirements. Sassy, notably, showed more favorable runtime than indelfree_bruteforce despite being an exhaustive edit-distance method, demonstrating that dedicated algorithmic optimizations can substantially improve the practical feasibility of exhaustive approaches. These resource constraints directly affects feasibility of current and future viromics studies, as spacer database size has been rapidly increasing—from 366 799 unique spacers in 2017 (Shmakov *et al.* 2017) to 1 173 006 unique spacers reported in 2021 (Dion *et al.* 2021) to 3 835 942 unique spacers in 2023 (Camargo *et al.* 2023). Similarly, public virus and MGE databases are growing rapidly, with large contributions from metagenomic samples resulting in routine fold increases in the number of predicted viral contigs (Camargo *et al.* 2023).

### Biological interpretation of spacer-protospacer matches

Beyond tool performance, the biological interpretation of identified matches requires careful consideration of several confounding scenarios. Low-complexity regions, whether in the viral target set or in the spacer set itself (where non-CRISPR repeated sequences may occasionally be misclassified as spacers), can produce spurious matches independently of tool choice. While certain tools employ internal filtering heuristics, a separate complexity masking step using tools such as DUST (Morgulis *et al.* 2006) or similar approaches (e.g. ldust, BBDuk) prior to searching remains advisable. In our analysis, we applied light filtration to the spacer database used in iPHoP, but observed that even a “cleaned” spacer set (name of sourced file) may contain a handful of very low complexity sequences.

Horizontal gene transfer (HGT) can spread conserved sequences across unrelated MGEs, creating genuine sequence similarities that do not reflect direct host–MGE interactions. Kosmopoulos *et al.* described a case where a transposon-mediated transfer of a phage lysin gene to the host genome produced a verifiable sequence match (Kosmopoulos *et al.* 2023), illustrating that an unknown fraction of observed alignments may reflect HGT rather than CRISPR-mediated interactions. Self-targeting events, where spacers match host genomic sequences, have been estimated to have putative exogenous origin (e.g. prophages) in approximately 50% (Stern *et al.* 2010) to 80% (Shmakov *et al.* 2017) of cases, though the rate of true non-defense self-targeting appears to be low (Shmakov *et al.* 2017). Additional complexities include CRISPR systems encoded on non-chromosomal replicons such as plasmids (Maier *et al.* 2019, Zhang *et al.* 2025), inter-species spacer acquisition as described in some archaea (Turgeman-Grott *et al.* 2019), and phage-encoded mini-arrays that can interfere with host CRISPR immunity (Shmakov *et al.* 2023).

The source and context of spacer data also merits consideration. Spacers extracted from assembled CRISPR arrays carry additional information—notably their position within the array and the observation that co-located spacers originate from the same host. Mitrofanov *et al.* (2025) identified system subtype-dependent patterns of spacer loss, and Vink *et al.* (2021) reported that most matched spacers in their analysis contained three or fewer mismatched nucleotides, consistent with our general recommendation of hamming distance ≤3. Vink *et al.* also observed strand-targeting preferences varying by CRISPR subtype (e.g. Type I-E and Type II systems preferentially target template strands), information that could enhance post-search verification when combined with sequence orientation and coding potential analysis.

### Study limitations

Several aspects of this study merit explicit acknowledgment of limitations. The synthetic datasets, while configured to match length distributions and nucleobase frequencies of real data (Supplementary Fig. S4), do not fully capture all structural features of biological sequences, including locus-specific composition biases, coding constraints, and evolutionary signatures. The approximately 2.5-fold higher normalized non-planned match rates observed in the semi-synthetic dataset relative to the fully synthetic simulations (Table 2) likely reflect such compositional features in real spacer sequences, and the non-planned match rates reported here should accordingly be interpreted as order-of-magnitude guides rather than precise predictive values.

**Table 2:** Dataset characteristics.

Dataset type	**Spacers (Count)**	**Contigs (Count)**	**Notes**
Fully Synthetic	100k–500k	5k–100k	Generated with varying params (see Table S4)
Semi-Synthetic	∼3.8M (Real)	∼420k (Simulated)	Real iPHoP spacers, Fully synthetic contigs matching IMG/VR4 GC% and length range.
Real Data (IMG/VR4)	∼3.8M (Real)	∼420k (Real “HQ”)	IMG/VR4 v1.1 HQ contigs and their subsamples, lightly filtered iPHoP spacers

**Table 3:** Comparison of non-planned match rates at hamming distance ≤3 between fully synthetic simulations and the semi-synthetic dataset.

	Fully-simulated (mean ± SD, *n* = 4)	Semi-synthetic (real spacers)	Ratio
Non-planned matches (total)	91.3 ± 64.5	56 273	n/a (different search space)
Per spacer per Gbp	0.0013 ± 0.0001	0.00068	0.53×
Per Gbp2 search space	40 314 ± 2669	20 239	0.50×

Exhaustive tools (Sassy, indelfree.sh bruteforce) could not be run on the full HQ benchmark dataset due to computational constraints. Our comparisons on the largest datasets therefore rely on the aggregate of heuristic tool results as a proxy for the complete positive set. For the same reason, edit-distance-based exhaustive analysis was limited to the smaller fully synthetic datasets. This tool-aggregate should therefore be considered as an underestimate for the actual number of valid alignments.

Another caveat lies in our current focus on hamming distance with regards to simulated spacer occurrences. This means that insertions/deletions are essentially ignored for most of our analysis. While mutations related to CRISPR targeting are broadly expected to be substitutions and indels have not been reported in this context, this lack of reports does not equate lack of the phenomena occurring, and we have enabled the option for the simulated spacers to vary in frequency and type of mutations. We hope this would enable future versions of the benchmark to estimate different tools’ ability to identify these cases, should they be established.

Additionally, tools were configured to maximize recall to the best of our ability, based on tool documentation, github issue threads and similar information sources. However, it is possible we either misconfigured certain tools, or that exhaustively exploring all possible parameter combinations would have yielded different results.

We also note that our analyses focused on assembled contigs from Illumina-derived data; for other sequencing technologies or raw-read-based workflows, additional considerations regarding sequencing error profiles would apply.

## Conclusion

This work demonstrates that tool choice in spacer-protospacer matching carries measurable consequences for downstream biological inference. Based on the empirical evidence presented, we suggest Bowtie1 at hamming distance ≤3 as a practical approach for large-scale analyses of host–MGE interactions through CRISPR spacers, given its combination of high sensitivity, computational efficiency, and consistency across dataset scales. For analyses requiring tolerance beyond 3 substitutions, indelfree.sh in indexed mode extends the hamming distance range without incurring a considerable elevated non-planned match rates, such as those observed for edit distance above 3. Of note, exhaustive tools (such as Sassy for edit and indelfree bruteforce) remain appropriate for small scale analyses—including experimental studies of individual host-phage systems (e.g. one or several phages and spacers from several arrays), or even medium-scale analysis (e.g. lineage-specific comparison such as all arrays from a specific bacterial genus and all their known phages). Additionally, the exhaustive tools can simplify result validation of heuristic tool outputs—where the total subject sequence is modest or if computational resources permit complete enumeration. For large scale analysis, such as using complete public spacer and large virus databases, a combination of heuristic tools currently appears as the only possible option.

Edit distance metrics may be warranted under specific conditions: when analyzing low-accuracy long-read data where sequencing-induced indels cannot be excluded, when investigating escape mutation mechanisms in controlled experimental systems where the mutation type itself is informative (and could be verified experimentally post-hoc), or when working in gene-editing off-target prediction contexts where gapped alignments have established biological relevance (such as RNA structure importance for activity). For all other cases, hamming distance ≤3 appears to capture the dominant mode of protospacer divergence (substitutions) as currently reported in literature, while maintaining a favorable ratio of genuine to non-planned matches. We emphasize that regardless of tool choice, post-hoc verification of alignment quality, complexity filtering, and contextual biological analysis remain important components of any spacer-protospacer matching workflow in the context of host range prediction.

## Supplementary Material

btag394_Supplementary_Data

## Data Availability

All code generated for this study can be found in the git repository: github.com/UriNeri/spacer_matching_bench. All data generated in this project (tool results and the analysed datasets, any sequence data, SLURM logs etc) are available on Zenodo https://doi.org/10.5281/zenodo.15171878.
