# Supplementary material for: Computational tool choice impacts CRISPR spacer-protospacer detection
Source: Bioinformatics. 2026 Jun 29;42(7):btag394. doi: 10.1093/bioinformatics/btag394 (PMC13326750; doi:10.1093/bioinformatics/btag394)
Supplement: btag394_Supplementary_Data [file btag394_supplementary_data.zip › supplementary_legend.pdf]

Supplementary Legends for: Computational Tool Choice Impacts CRISPR Spacer-Protospacer Detection

Uri Neri   Antonio Pedro Camargo   Rick Beeloo   Brian Bushnell   Simon Roux

1 Supplementary Materials

1.1 Tables

1.1.1 Supplementary Table S1: Tool Configuration Details

`supp_table_S1_tool_config.tsv`

Parameters chosen for each tool to maximize recall on short spacers. BLASTn settings emphasize ungapped alignments and permissive identity/coverage to capture all valid matches; bowtie variants, minimap2, MMseqs2, mummer4, sassy, strobealign, and x_mapper entries list versions and command-line templates.

1.1.2 Supplementary Table S2: IMG/VR4 Contig Dataset Statistics and Filtering Steps

`supp_table_S2_contig_stats.tsv`

Summary of IMG/VR4 v1.1 high-confidence viral contigs after taxonomic filtering and length cutoffs, including the final HQ benchmark subset (fraction_1).

1.1.3 Supplementary Table S3: CRISPR Spacer Dataset Composition and Statistics

`supp_table_S3_spacer_stats.tsv`

Statistics for the iPHoP spacer dataset before and after complexity filtering, including length ranges, GC content, and removal criteria.

1.1.4 Supplementary Table S4: Detailed Statistics for All Datasets

`supp_table_S4A_real_dataset_sequence_stats.tsv`

A. Real datasets — Counts and length/GC summaries for spacers and contigs across IMG/VR4 fractions (0.0005 through 1).

`supp_table_S4B_simulated_dataset_sequence_stats.tsv`

B. Simulated datasets — Equivalent summaries for all synthetic datasets used in the study.

1.1.5 Supplementary Table S5: Simulation and job-generation configuration

`supp_table_S5_prepare_jobs.tsv`

Per-dataset group configuration for simulations and tool runs, noting search-space sizes, distance thresholds, and tool inclusion/exclusion.

1.1.6 Supplementary Table S6: Recall Values for Each Tool at Different Mismatch Thresholds

`supp_table_S6_recall_values.tsv`

Values are counted per spacer–contig alignment using exact distance for each metric. `n_found` tallies tool-reported positive alignments at a given distance, `n_total` is the ground-truth positive count, and recall is `n_found / n_total`. Subsections report IMG/VR4 and synthetic datasets separately. Note that these are aggregated per unique `alignment_idx`, not per unique `region_idx` (i.e. without applying the boundary tolerance and per-region aggregations). The data and results displayed in the main text refer to the per-region aggregate. The entire results (detailing which alignment were detected by which tools and to which `region_idx` they belong) can be regenerated using the projects' analysis notebook and the raw tool outputs (available in the Zenodo deposit, see Data availability).

1.1.7 Supplementary Table S7: Search-space-normalized metrics at different hamming/edit distances

`supp_table_S7_search_space_normalized.tsv` Normalized non-planned match rates (per spacer per Gbp and per Gbp^2) for each fully synthetic simulation.

1.1.8 Supplementary Table S8: Semi-synthetic non-planned match rates by hamming threshold

`supp_table_S8_semi_synthetic_rates_hamming_background.tsv`

Cumulative non-planned matches in the semi-synthetic dataset across hamming thresholds (0–5), with rates normalized by spacer and contig sequence space. Note: For the higher thresholds (4, 5) the counts are should be taken as lower bounds as only some of the heuristic tools could finish within reasonable time. Furthermore, so that some of the tools could finish within said time limit, we optimised the parameters to a maximum value of 3 mismatches (incidentally the hard limit of Bowtie1).

1.1.9 Supplementary Table(s) S9: Computational Resource Usage

Detailed resource usage metrics for all completed tool runs on both real (IMG/VR4 subsampled) and simulated datasets. For each tool-dataset combination, we report total CPU time (hours), wall-clock time (hours), peak memory usage (GB), SLURM job ID, and allocated CPU cores. All tools were allocated 64 threads and 512 GB RAM on identical hardware (dual AMD EPYC 7543, see Methods the Reproducibility section in the main text). The fraction_X datasets represent subsampled fractions of IMG/VR4 HQ contigs, while ns_X_nc_Y datasets are synthetic benchmarks. The ns_3826979_nc_421431_real_baseline dataset is semi-synthetic, where real spacers are searched against simulated contigs matched to IMG/VR4 characteristics.

1.1.9.1 A. Real data (IMG/VR4 subsampled fractions) — completed jobs

`supp_table_S9A_real_data_resource_usage.csv`

1.1.9.2 B. Simulated datasets — completed jobs

`supp_table_S9B_simulated_data_resource_usage.csv`

1.1.9.3 C. Timed-out / failed jobs

`supp_table_S9C_timed_out_failed_jobs.csv`

1.2 Supplementary Figures

1.2.1 Supplementary Figure S1: IMG/VR4 tool-vs-tool comparison matrices

`FigureS1_supp_imgvr4_fraction1_tool_comparison_matrix.svg`

Pairwise tool-comparison matrices for IMG/VR4 fraction_1 at exact hamming distances (0–3). Each cell indicates regions detected by one tool but not another; diagonals list per-tool totals.

1.2.2 Supplementary Figure S2: Simulated dataset recall vs spacer occurrence frequency

`FigureS2_supp_imgvr4_high_ins_recall_vs_occurrence.svg`

High-occurrence-focused recall view for the high-insertion simulated dataset (`ns_500_nc_5000_HIGH_INSERTION_RATE`) at hamming distance 3.

1.2.3 Supplementary Figure S3: IMG/VR4 fraction_1 recall vs occurrence frequency at hamming distance 3

`FigureS3_supp_imgvr4_fraction1_recall_vs_occurrence.png`

Recall versus spacer occurrence for the real IMG/VR4 fraction_1 dataset at hamming distance 3.

1.2.4 Supplementary Figure S4: Comparison of simulated vs real spacer characteristics

`FigureS4A_supp_filtered_iphop_spacers_attribute_distributions_multiplot.png` `FigureS4B_supp_simulated_spa`

Distributions comparing simulated and real spacer features (k-mer repeatability, entropy, base frequencies, GC, LCC). Panel A shows filtered iPHoP spacers; Panel B shows fully simulated spacers (ns_100000_nc_20000).

1.2.5 Supplementary Figure S5: Spacer characteristic distributions by dataset

`FigureS5_supp_real_data_contig_distributions.svg`

Spacer and contig characteristic distributions for real (IMG/VR4) and simulated datasets, covering spacer size, mismatch counts, and occurrence rates. Outliers were removed for fraction_1 length visualization.

1.2.6 Supplementary Figure S6: Distribution of matched spacers from fraction_1

`FigureS6_supp_fraction_1_matched_spacers_distributions.png`

Distributions of matched spacers (hamming 3) across length, number of occurrences, and per-distance alignment counts.

1.2.7 Supplementary Figure S7: Peak memory scaling (real and simulated datasets)

`FigureS7_supp_resource_usage_memory_2panel.svg`

Peak memory usage (RSS) scaling with dataset size. Axes are log-scaled; tool identity encoded by color/shape.

Panel A: IMG/VR4 subsamples;

Panel B: simulated datasets.

1.2.8 Supplementary Figure S8: Tool recall consistency across dataset sizes

`FigureS8A_supp_imgvr4_across_fractions_recall_consistency_hamming_le3.svg` `FigureS8B_supp_simulated_cross`
`FigureS8C_supp_simulated_heatmap_dataset_consistency_hamming_le3.svg`

Per-tool recall consistency at hamming distance 3. Panel A: real IMG/VR4 subsamples. Panel B: synthetic datasets. Panel C: heatmap of per-tool recall across simulated runs.

1.2.9 Supplementary Figure S9: Per-simulation non-planned match counts (Hamming vs Edit)

Per-simulation comparisons of non-planned match counts under hamming versus edit distance for four fully synthetic datasets (`ns_50000_nc_5000`, `ns_75000_nc_5000`, `ns_100000_nc_10000`, `ns_100000_nc_20000`) at thresholds 0–5.

`FigureS9A_supp_ns_50000_nc_5000_background_counts_foldchange.svg` `FigureS9B_supp_ns_75000_nc_5000_backgro`
`FigureS9C_supp_ns_100000_nc_10000_background_counts_foldchange.svg` `FigureS9D_supp_ns_100000_nc_20000_bac`

Grouped bars for each simulation (`ns_50000_nc_5000`, `ns_75000_nc_5000`, `ns_100000_nc_10000`, `ns_100000_nc_20000`) comparing hamming and edit distance non-planned match counts at thresholds 0–5.

1.3 Supplementary Notes

1.3.1 Supplementary Note 1: Coordinate Tolerance Matching Example

To illustrate the necessity for coordinate tolerance matching, consider the following case from one of the synthetic dataset (`ns_100000_nc_10000`) where three different tools detected the same ground truth region, but reported it with slightly different coordinates:

Source	Spacer ID	Contig ID	Query Start	Query End	Target Start	Target End	Strand	Mismatches/Cost (tool-reported)	Notes
Ground Truth	1c47e31b_spacer_1_0034	1c47e31b_contig_1828	-	-	5001	5036	reverse	1	
BLASTn	1c47e31b_spacer_1_0034	1c47e31b_contig_1828	1	34	5036	5003	reverse	0	
MMseqs2	1c47e31b_spacer_1_0034	1c47e31b_contig_1828	1	35	5036	5002	reverse	1	
Sassy	1c47e31b_spacer_1_0034	1c47e31b_contig_1828	-	-	5000	5036	reverse	1	CIGAR: 34=1I1=

In this example, BLASTn reports the ungapped alignment at contig positions 5003-5036 (0 reported mismatches), MMseqs2 reports 5002-5036 (1 reported mismatch over the terminal base), and Sassy reports 5000-5036 (1 reported insertion, suggesting the position 5000 in the contig matches that of the last position in the spacer). The ground truth planned occurrence was at 5001-5036. Despite these coordinate variations, all three tools detected the same biological match. **Critically**, all three tools report alignments that are acceptable matches to the ground truth occurrence, under a maximum hamming distance of 1. Via this coordinate tolerance, we avoid "double-counting" when aggregating results across tools.

1.4 References and Data Availability

Analysis notebooks and source code is available in the project repository: https://github.com/UriNeri/spacer_matching_benc
The raw tool results, the simulated datasets, and the fraction of real datasets, have been uploaded to the project's zenodo deposit (DOI: 10.5281/zenodo.15171878)
